# Precios, disponibilidad y asequibilidad de insulina en farmacias públicas y privadas en Perú

**DOI:** 10.26633/RPSP.2019.85

**Published:** 2019-10-31

**Authors:** Janeth Tenorio-Mucha, María Lazo-Porras, Liliana Hidalgo-Padilla, David Beran, Margaret Ewen

**Affiliations:** 1 Centro de Excelencia en Enfermedades Crónicas, CRONICAS Universidad Peruana Cayetano Heredia Lima Perú Centro de Excelencia en Enfermedades Crónicas, CRONICAS, Universidad Peruana Cayetano Heredia, Lima, Perú.; 2 Departamento de Medicina Tropical y Humanitaria Universidad de Ginebra y Hospitales Universitarios de Ginebra Ginebra Suiza Departamento de Medicina Tropical y Humanitaria, Universidad de Ginebra y Hospitales Universitarios de Ginebra, Ginebra, Suiza.; 3 Acción Internacional para la Salud Acción Internacional para la Salud Ámsterdam Países Bajos Acción Internacional para la Salud, Ámsterdam, Países Bajos.

**Keywords:** Insulina, metformina, acceso a medicamentos esenciales y tecnologÍas sanitarias, precio de medicamento, Perú, Insulin, metformin, access to essential medicines and health technologies, drug price, Peru, Insulina, metformina, acesso a medicamentos essenciais e tecnologias em saúde, preço de medicamento, Peru

## Abstract

**Objetive.:**

Medir el precio, disponibilidad y asequibilidad de insulina y metformina, como comparador, en farmacias públicas y privadas en seis regiones del Perú.

**Métodos.:**

Estudio transversal con uso de la metodología adaptada de la Organización Mundial de la Salud/Acción Internacional para la Salud (OMS/AIS). Se encuestaron farmacias públicas y privadas de seis regiones del Perú. Se recolectaron datos de disponibilidad y precio de insulina (todos los tipos) y metformina en presentación de 850 mg. La disponibilidad se expresa en porcentajes y los precios se reportan en medianas. La asequibilidad se define como el número de días que debe laborar una persona con el salario mínimo para cubrir el costo de un mes de tratamiento.

**Resultados.:**

La disponibilidad en farmacias públicas es de 63,2% para insulina regular y 68,4% para isófana-NPH, pero se observaron diferencias de disponibilidad entre los niveles de atención y entre las regiones. En farmacias privadas, la variedad de insulina es mayor, pero la disponibilidad es menor del 11%. La mediana de precios para la insulina humana en farmacias privadas fue entre tres a cuatro veces mayor que en farmacias públicas. En comparación, la disponibilidad de metformina alcanza 89,5% en farmacias públicas y 77,7% en privadas. La asequibilidad en farmacias públicas para un mes de tratamiento con insulina humana o metformina genérica es menor a lo percibido por un día laborable.

**Conclusiones.:**

El precio de insulinas humanas y de metformina genérica en farmacias públicas es asequible. Sin embargo, se necesitan esfuerzos para mejorar su disponibilidad en las regiones y los niveles de atención.

## Abstract (en)

**Objective.:**

Measure and compare the price, availability, and affordability of insulin and metformin in public and private pharmacies in six regions of Peru.

**Methods.:**

Cross-sectional study using the World Health Organization/Health Action International (WHO/HAI) revised methodology. Public and private pharmacies in six regions of Peru were surveyed. Data were collected on availability and prices for insulin (all types) and 850 mg metformin. Availability is expressed as percentages and prices are reported in medians. Affordability is defined as the number of days that a person must work at minimum wage to cover the cost of one month of treatment.

**Results.:**

Availability in public pharmacies is 63.2% for regular insulin and 68.4% for NPH (isophane) insulin, but differences in availability were observed between levels of care and between regions. Private pharmacies have a greater variety of insulin, but availability is less than 11%. The median price of human insulin was three to four times higher in private pharmacies than in public pharmacies. In comparison, availability of metformin was 89.5% in public pharmacies and 77.7% in private ones. Affordability in public pharmacies for one month of treatment with human insulin or generic metformin is less than one day’s wages.

**Conclusions.:**

The price for human insulin and generic metformin in public pharmacies is affordable. However, efforts are needed to improve their availability in the different regions and levels of care.

## RESUMO

**Objetivo.:**

Avaliar o preço, a disponibilidade e a acessibilidade à insulina e metformina, como comparativo, em farmácias públicas e privadas em seis regiões do Peru.

**Métodos.:**

Estudo transversal conduzido com metodologia adaptada da Organização Mundial da Saúde/Ação Internacional para a Saúde (OMS/AIS). Farmácias públicas e privadas foram pesquisadas em seis regiões do Peru com a coleta de dados sobre a disponibilidade e os preços da insulina (de todos os tipos) e metformina na apresentação de 850 mg. A disponibilidade é expressa em porcentagem e os preços estão descritos como mediana. A acessibilidade foi definida no estudo como o número de dias que um indivíduo que ganha salário mínimo precisa trabalhar para arcar com o custo de um mês de tratamento.

**Resultados.:**

A disponibilidade em farmácias públicas da insulina regular foi de 63,2% e da insulina isófana (NPH) foi de 68,4%, porém se observou diferença de disponibilidade entre os níveis de atenção e as regiões. Nas farmácias privadas, a variedade de insulinas é maior, mas a disponibilidade é inferior a 11%. A mediana de preços para a insulina humana nas farmácias privadas é 3 a 4 vezes maior que nas farmácias públicas. Comparativamente, a disponibilidade da metformina foi de 89,5% nas farmácias públicas e 77,7% nas farmácias privadas. A acessibilidade nas farmácias públicas para um mês de tratamento com insulina humana ou metformina genérica é inferior à remuneração de um dia de trabalho.

**Conclusões.:**

As insulinas humanas e a metformina genérica têm preço acessível nas farmácias públicas. Porém, esforços devem ser empreendidos para aumentar a disponibilidade destes medicamentos nos níveis de atenção e nas regiões.

La diabetes mellitus (DM) es un problema para la salud pública mundial, contribuye a la carga de enfermedad con 2,7% de años de vida ajustados por discapacidad (AVAD) a nivel mundial y con 4,6% de AVAD en países de América Latina y el Caribe (ALC) (1). En 2018, la DM contribuyó a la carga de enfermedad con 2,9% de AVAD en Perú (1). Según estudios realizados en el 2012, la prevalencia de DM en la población peruana fue de 7% (2), 96,8% del total de casos corresponden a DM de tipo 2 y 2,5% a DM de tipo 1 (3). La insulina es un medicamento imprescindible para el tratamiento de la DM de tipo 1 y, en ocasiones, necesaria para el tratamiento de la DM de tipo 2 (4). Un análisis del sistema de salud peruano reportó que aproximadamente 30% de los pacientes con DM de tipo 2 recibe prescripción de insulina sola o combinada con hipoglucemiantes orales como la metformina (5, 6).

En 2011, debido a la creciente carga de enfermedad por enfermedades no transmisibles (ENT), la Asamblea General de la Naciones Unidas para la Prevención y Control de Enfermedades No Transmisibles invocó a mejorar el acceso a medicamentos, fortalecer los sistemas de salud y garantizar la cobertura de salud para atender de manera prioritaria las ENT, entre ellas la diabetes (7). Además, en la Agenda de Desarrollo Sostenible para el 2030, se aprobó disminuir la mortalidad prematura por ENT en un tercio (8). Para lograrlo, es preciso que los pacientes ejerzan su derecho de acceso a medicamentos esenciales (9).

Las barreras en el acceso a medicamentos para ENT son multifactoriales e involucran a todo el sistema de salud (10). Datos de 36 países de ingresos bajos y medianos (PIBM) (11) revelan que, aunque en muchos países el acceso a medicamentos en el sector público es gratuito, la disponibilidad es baja. Mientras, el sector privado ofrece mayor variedad de tipos de insulina, aunque con precios inasequibles (11). Según los datos de precios y disponibilidad recolectados de 15 PIBM, la disponibilidad promedio de insulina fue de 56% y 37% en establecimientos del sector público y privado, respectivamente (4).

A pesar de la complejidad e impacto del problema, el análisis del precio, disponibilidad y asequibilidad de medicamentos para la diabetes es un tema que aún no ha sido abordado de manera adecuada en Perú. Un estudio realizado en Lima, Perú, en el 2013 (6), reportó que la insulina solo se encontraba disponible en hospitales del tercer nivel de atención y farmacias privadas, y que los precios de insulina resultaron hasta tres veces más altos en farmacias privadas en comparación con las farmacias públicas (6). Sin embargo, la metodología de este estudio no proporcionó suficiente información de otras áreas del Perú, fuera de la capital.

Por las consideraciones anteriores, es importante proveer un mejor entendimiento de la situación, para compararla con otros países e idear intervenciones que mejoren el acceso al tratamiento. El presente estudio propone medir el precio, disponibilidad y asequibilidad de insulina y metformina en seis regiones del Perú. Este estudio recibió aprobación por el Comité Institucional de Ética de la Universidad Peruana Cayetano Heredia, del Hospital Arzobispo Loayza y del Hospital Dos de Mayo. El Ministerio de Salud autorizó la realización del estudio en las farmacias públicas y se coordinó con cada hospital el horario de visitas. Las personas encargadas en las farmacias privadas dieron su consentimiento para ser incluidas en forma anónima en el estudio.

## MATERIALES Y MÉTODOS

El estudio se realizó en la capital del país (Lima), así como en otras cinco regiones del Perú con mayor población, que se encontraban como máximo a un día de viaje de distancia (Arequipa, Ayacucho, Junín, La Libertad y San Martín). En el cuadro 1 se muestran algunas de las características de cada región. Se trata de un estudio transversal, realizado entre los meses de abril y mayo del 2018, con la metodología adaptada de la Organización Mundial de la Salud/Acción Internacional para la Salud (OMS/AIS) (12). Se recolectaron datos del precio y disponibilidad de insulina y metformina en farmacias públicas y privadas.

Perú cuenta con proveedores de salud públicos y privados organizados de manera segmentada (13, 14). Para este estudio, las farmacias públicas están representadas por farmacias hospitalarias del segundo y tercer nivel de atención del Ministerio de Salud y servicios de salud municipales (MINSA-GR), y las farmacias privadas están representadas por farmacias comunitarias independientes o pertenecientes a grupos comerciales (cadenas). El MINSA-GR provee medicamentos de manera gratuita a la población afiliada al Seguro Integral de Salud (SIS), pero también ofrece medicamentos a la población no asegurada a cambio del pago del bolsillo (13). Por su parte, en las farmacias privadas, la adquisición de medicamentos es por compra directa.

Primero se muestrearon farmacias públicas: en cada región se seleccionó una farmacia de un hospital regional y cuatro de hospitales provinciales o distritales a una distancia de entre tres a cuatro horas del hospital regional. Luego, se escogió la farmacia privada más cercana a cada farmacia pública. Solo se podían seleccionar aquellas farmacias con licencia de funcionamiento vigente y autorizadas por la Agencia Reguladora de Medicamentos. Para su selección, se utilizó el Registro Nacional de Establecimientos Farmacéuticos de la Agencia Reguladora de Medicamentos.

Además, se muestrearon farmacias de reserva, que fueron encuestadas cuando las muestreadas en la primera tanda rehusaron participar o cuando, durante la recolección de datos, no proporcionaron información de al menos uno de los medicamentos en estudio. La encuesta en las farmacias de reserva permitió asegurar una cantidad suficiente de datos de precios para un análisis robusto. Para no afectar la precisión de los resultados de disponibilidad, los datos de las farmacias de la muestra primaria se incluyeron en el análisis de disponibilidad.

En total, se seleccionó una muestra primaria de 60 farmacias, 5 por cada sector (público o privado) y región, que es el número mínimo de establecimientos que indica la metodología de la OMS/AIS (12). Durante la conducción del estudio, fue necesario visitar farmacias de reserva en las regiones de San Martín, Ayacucho, La Libertad y Lima debido a que no se encontró insulina en los establecimientos encuestados en primer lugar (cuadro 1).

Antes de las visitas, se entrenó al personal de campo y se realizó un piloto en una farmacia privada que no formó parte de la muestra primaria. Para la recolección de datos, se visitaron los establecimientos en parejas y se solicitaron los productos para comprobar su disponibilidad.

Para la insulina, se recogieron datos de todas las formas disponibles, humanas y análogas, incluida información del tipo, nombre de marca, fabricante, importador, concentración (UI/mL), forma farmacéutica (vial, cartucho o lapicero precargado), cantidad (mL), unidades por paquete y precio por paquete. Para el tipo de registro, se consideró como originales a las insulinas fabricadas por Sanofi (Gentilly, Francia), Eli Lilly (Indianápolis, Estados Unidos de América) y Novo Nordisk (Bagsværd, Dinamarca).

Para la metformina en su presentación de 850 mg, se recogieron datos de Glucophage^®^, medicamento original de marca, así como del genérico de precio más bajo (GPB) disponible en el establecimiento. Se recogieron datos de disponibilidad, nombre de marca, fabricante, tamaño del paquete y precio por paquete.

Cada supervisor de área validó la recolección de datos, y volvió a aplicar la encuesta en una farmacia privada por cada región visitada. Los datos se ingresaron en planillas de cálculo en Excel^®^ por duplicado y luego se los comparó para verificar datos incompletos o inconsistencias.

Los análisis se realizaron según las definiciones propuestas por la OMS/AIS (12). Los cálculos se realizaron en Microsoft Excel 2016^®^ y Stata v.14^®^ (Stata Corp, College Station, Texas, EE.UU.).

**CUADRO 1.** Características de las regiones encuestadas y número de farmacias encuestadas por región en Perú, 2018

Regiones	Características de las regiones			Farmacias encuestadas			
	Población total^a^ (habitantes)	Población rural^b^ (%)	Ubicación geográfica	Farmacias públicas		Farmacias privadas	Total
				2° nivel	3° nivel		
Lima	10 143 003	2	Costa central	4	3	18	25
La Libertad	1 905 301	22	Costa norte	6	1	25	32
Arequipa	1 287 205	10	Costa/sierra sur	3	2	9	14
Junín	1 370 274	34	Sierra central	5	1	12	18
San Martín	862 822	35	Selva norte	6	NA	26	32
Ayacucho	703 629	45	Sierra sur	7	NA	31	38
	TOTAL			31	7	121	159

a Instituto Nacional de Estadística e Informática (INEI): estimaciones y proyecciones de población, 2017.

b INEI: crecimiento y distribución de la población, 2017. Primeros resultados.

### Disponibilidad

Se expresa como el porcentaje de farmacias en las cuales estuvo disponible el medicamento al momento de la encuesta.

### Precio

Los precios ofrecidos al paciente se expresan en medianas. Para las insulinas, se informa el precio para dosis de 1000 UI y, para la metformina en presentación de 850 mg, se informa el precio para dosis individuales. El dato de precio se recogió en la moneda local, el sol (PEN) y se convirtió a dólares estadounidenses (USD) con base en el tipo de cambio del primer día de recolección de datos.

Según la cotización oficial del día 8 de abril de 2018, el cambio era 1 USD = 3232 PEN.

Además, se obtuvieron los datos del último proceso de compra centralizada por el gobierno (en el año 2016) para insulina humana regular, insulina isófana-NPH y metformina, se comparó con la mediana de precios ofrecida al paciente en las farmacias públicas y se calculó el porcentaje de incremento en el precio.

### Asequibilidad

La asequibilidad se ha expresado como el promedio del número de días que un trabajador con sueldo mínimo debería laborar para costear un tratamiento mensual, es decir, para dosis de 1000 UI (10 mL de 10 UI/mL) de insulina y 60 tabletas de metformina de 850 mg. El cálculo se realizó con base en el salario mínimo mensual en Perú, de 930 PEN (equivalentes a 287,60 USD (15).

**FIGURE 1.**
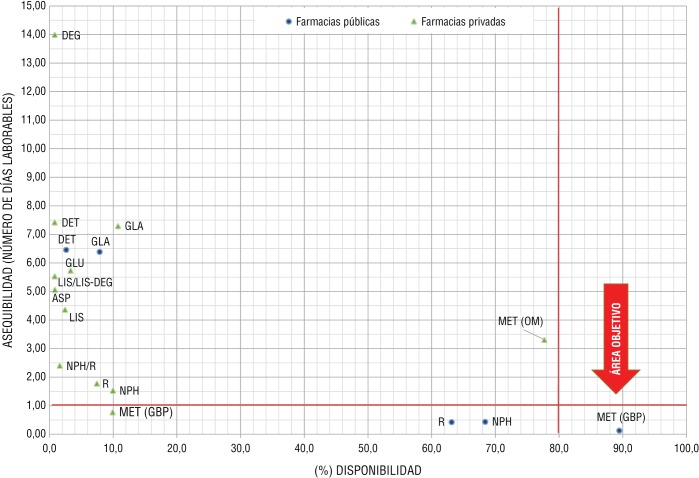
Disponibilidad y asequibilidad de medicamentos para la diabetes, Perú, abril-mayo, 2018

Además, se realizó un gráfico de tendencia de asequibilidad y disponibilidad, en el eje “x” se representó el porcentaje de disponibilidad para cada medicamento y en el eje “y” el promedio de asequibilidad de estos (figura 1). Luego se trazaron puntos de corte en 80% para disponibilidad y de 1 día para asequibilidad.

## RESULTADOS

En total, se recolectaron datos de 159 farmacias entre públicas y privadas. En el cuadro 1 se muestran las características principales de cada región y el número de farmacias encuestadas por región. Las características de las insulinas encontradas (n = 119) se muestran en el cuadro 2.

### Disponibilidad de insulina

En las farmacias públicas, se encontraron insulinas humanas disponibles en 63,2% y 68,4%, para insulina regular e isófana-NPH, respectivamente. Además, se encontraron insulinas análogas en 10,5% de las farmacias. La disponibilidad en el segundo nivel de atención para la insulina regular e isófana-NPH fue de 54,8% y 61,3%, respectivamente. En el tercer nivel de atención, para los mismos tipos de insulina, la disponibilidad alcanzó 100%. También se observaron diferencias de disponibilidad entre regiones (cuadro 3). En las farmacias privadas fue más probable encontrar mayor variedad de tipos de insulina (humana regular, 7,4%; humana isófana-NPH, 9,9%; mixta humana, 1,7%; análoga rápida, 5,8%; análoga prolongada, 10,7% y análoga mixta, 0,8%). Por otro lado, en las farmacias públicas se encontraron viales en 92,9% y biosimilares en 91,1%. En las farmacias privadas se hallaron viales en 54,0% y biosimilares en 39,7%.

### Precio de la insulina

En general, la mediana del precio pagado por los pacientes en farmacias privadas fue mayor que en las farmacias públicas para todos los tipos de insulina (cuadro 4). En las farmacias privadas, las insulinas humanas costaron entre 3 a 4 veces más que en las públicas.

En farmacias públicas, la mediana de precio y rango para insulina regular fue de 4,02 USD (rango: 2,78 - 10,21 USD) y, para insulina isófana-NPH, de 4,08 USD (rango: 3,40 - 12,56 USD). Los precios más altos se detectaron en los establecimientos del segundo nivel. La diferencia de precios en farmacias privadas fue más amplia (cuadro 4).

Los precios de adquisición del gobierno por compra centralizada correspondiente al año 2016 son de 3,59 USD para la insulina regular y de 3,66 USD para la insulina isófana-NPH. Se calculó un porcentaje de incremento de precio de 12,2% y 11,7% para la insulina regular e isófana-NPH, respectivamente, usando la mediana de precio de compra del paciente en farmacias públicas (cuadro 4). Además, se obtuvo el precio de compra institucional para la insulina glargina de 51,57 USD (con un incremento de precio de 18,7%) y, para la insulina detemir, de 52,08 USD (con un incremento de precio de 18,8%). El porcentaje de incremento corresponde a gastos por operaciones de gestión y distribución del medicamento a los almacenes y farmacias.

Elaboración propia a partir de los resultados del estudio. Se considera como área objetivo cuando la disponibilidad es > 80% y la asequibilidad es menor a un día laborable de un trabajador con el sueldo mínimo (287.60 USD) para adquirir 1000 UI de insulina o 60 tabletas de metformina.

R, insulina regular; NPH, insulina isófana-NPH; NPH/R, insulina mixta humana; ASP, insulina aspart; GLU, insulina glulisina; LIS, insulina lispro; GLA, insulina glargina; DET, insulina detemir; DEG, insulina deglutec; LIS/LIS-DEG, insulina mixta análoga; MET (GBPP), metformina 850 mg genérica de bajo precio, MET (OM), metformina 850 mg original de marca.

### Asequibilidad de la insulina

Respecto a la asequibilidad, en general, las insulinas son más asequibles en farmacias públicas que en farmacias privadas. En la figura 1 se observa que la asequibilidad de insulinas humanas en farmacias públicas es menor al equivalente a un día laborable y su disponibilidad está por debajo de 80%. Por otro lado, tanto la disponibilidad y asequibilidad de todos los tipos de insulinas en farmacias privadas son menores a 80% y mayores a un día laborable, respectivamente.

### Disponibilidad, precio y asequibilidad de la metformina

La disponibilidad de metfomina GPB en farmacias públicas alcanzó 89,5%, mientras que en farmacias privadas fue de 77,7%. La metformina original de marca alcanzó 9,9% de disponibilidad en farmacias privadas. Al igual que para insulina, la disponibilidad de metformina varía entre las regiones: mientras que en Lima alcanza 71,4%, en Ayacucho, La Libertad y San Martín llega a 100%.

La mediana de precio para la metformina en farmacias públicas es de 0,02 USD (rango: 0,02 - 0,21 USD) y en farmacias privadas para GPB es de 0,12 USD (rango: 0,03 - 0,31 USD), mientras que la original de marca cuesta 0,53 USD (rango: 0,42 - 0,81 USD). En la figura 1 se observa que la disponibilidad de la metformina GBP, tanto para farmacias públicas como para privadas, es mayor de 80%, y la asequibilidad es menor a un día laboral en ambos sectores.

## DISCUSIÓN

En general, existen diferencias de precio y disponibilidad entre los sectores (público y privado) y las regiones estudiadas. La disponibilidad en farmacias públicas fue de 63,2% para insulina regular y 71,1% para isófana-NPH, pero los valores varían entre las regiones y los niveles de atención. Respecto al precio de compra para los pacientes, la mediana del precio de la insulina humana en farmacias privadas, en comparación con el precio de farmacias públicas fue entre 3 y 4 veces más alta. Las insulinas análogas se encuentran, sobretodo, en farmacias privadas, pero a un mayor precio y menos asequibles que las insulinas humanas. En comparación, metformina está más disponible y asequible en ambos sectores.

En 2018, un estudio epidemiológico realizado en 22 países (16) sobre medicamentos para diabetes identificó *stocks* limitados y precios restrictivos, sobre todo en PIBM. Mientras que la metformina estuvo disponible en 100% de establecimientos en países de altos ingresos y la insulina en 94%. En los PIBM, estos valores fueron de 86,1% y 29,3%, respectivamente (17).

**CUADRO 2.** Características de las insulinas encontradas en las farmacias encuestadas en Perú, 2018

Detalle	n = 119	%
**Tipo de insulina**		
**Humanas**		
Regular	35	29,4
Intermedia, isófana-NPH	40	33,6
Mixta humana	2	1,7
**Análogas**				
Rápida
Glulisina	4	3,4
Lispro	3	2,5
Aspart	1	0,8
Prolongada		
Glargina	30	25,3
Detemir	2	1,7
Deglutec	1	0,8
Mixta análoga	1	0,8
**Fabricante**				
Wockhardt	68	57,1
Sanofi	25	21,0
Eli Lilly	12	10,1
Novo Nordisk	6	5,0
Jiangsu Wanbang Biochemical	6	5,1
Tongua Dongbao Pharmaceutical	2	1,7
**Presentación**				
Vial	86	72,3
Lapicero	33	27,7
**Origen**				
Biológico de referencia	43	36,1
Biosimilar	76	63,9

En época reciente, un estudio realizado con la metodología OMS/AIS, que recolectó datos en 13 PIBM encontró que la disponibilidad en el sector público fluctuó entre 55% y 80% y solo Brasil, Kirguistán y Pakistán (16) alcanzaron la meta de 80% del Plan de Acción Global para la prevención de enfermedades crónicas (18). Por otra parte, la disponibilidad de insulina en el sector privado fue de 45-53% para insulinas humanas y 27-36% para insulinas análogas. La mediana de precio de insulina en el sector público estuvo entre 8,78 USD y 10,15 USD para insulinas humanas y 31,3 USD y 47,5 USD para insulinas análogas. En farmacias privadas, el precio variaba entre 8,4 USD a 14,5 USD para insulinas humanas y 33,1 USD a 60,1 USD para insulinas análogas (16). En comparación con nuestros resultados, la disponibilidad de insulinas humanas a nivel nacional no alcanza la meta de 80%; además, existe variación de disponibilidad de insulina, tanto en las áreas encuestadas como en los sectores (público y privado).

El problema de la disponibilidad y asequibilidad bajas de insulina es complejo a nivel global y se atribuye a diferentes factores, entre ellos: mercado dominado por multinacionales, debilidades en la infraestructura para el abastecimiento, cadena de suministro y distribución, adquisición de insulina a través de importaciones que dependen de la autorización de comercialización; diferencias en el acceso a insulina a nivel nacional e internacional y, por último, el mantenimiento de la cadena de frío que requiere su conservación (19).

Creemos, además, que un problema clave en la disponibilidad de insulinas en Perú ocurre durante la estimación de los requerimientos de medicamentos para la diabetes, pues hay un registro impreciso de la cantidad de pacientes con diabetes atendidos en los hospitales públicos. Además, los datos utilizados para la estimación se basan en el consumo histórico de los meses anteriores (20, 21). Sin embargo, el consumo histórico no refleja la demanda real, pues no tiene en cuenta las recetas que se dispensan de forma incompleta o que no se dispensan en absoluto, por lo que se subestima el requerimiento.

**CUADRO 3.** Disponibilidad de insulina en farmacias públicas y privadas por cada región en Lima, 2018

Región	Farmacias	Tipo de insulina					
		Humana			Análoga		
		Regular (%)	Isófana-NPH (%)	Mixta (%)	Rápida (%)	Prolongada (%)	Mixta (%)
Lima	Públicas	85,7	85,7	NA	NA	14,3	NA
	Privadas	11,1	33,3	NA	11,1	27,8	NA
La Libertad	Públicas	42,9	14,3	NA	NA	14,3	NA
	Privadas	8,0	4,0	NA	4,0	8,0	NA
Arequipa	Públicas	1,0	100,0	NA	NA	NA	NA
	Privadas	11,1	11,1	11,1	11,1	22,2	11,1
Junín	Públicas	83,3	83,3	NA	NA	16,7	NA
	Privadas	NA	NA	NA	NA	NA	NA
San Martin	Públicas	50,0	83,3	NA	NA	NA	NA
	Privadas	15,4	11,5	3,8	1,5	15,4	NA
Ayacucho	Públicas	28,6	57,1	NA	NA	14,3	NA
	Privadas	NA	3,2	NA	NA	NA	NA

Por otro lado, en Perú, la diferencia en los precios depende del tipo de compras de medicamentos que realizan los hospitales. Existe un mecanismo de compras centralizadas en las que participan varios establecimientos de salud a nivel nacional y permiten tomar ventaja de las economías de escala y conseguir menores precios. Sin embargo, también es posible realizar compras descentralizadas y son estas las que pagan precios más altos por la compra de insulinas, en especial con la compra de análogos. Algunas soluciones para el pago de precios muy elevados por la compra de insulina, sería centralizar el abastecimiento de insulinas humanas, revisar los procesos de compra de medicamentos y eliminar los requisitos que impactan de forma negativa en el abastecimiento de insulina (16).

**CUADRO 4.** Mediana de precios por tipo de insulina para farmacias públicas y privadas (1000 IU)^a^, Perú, abril-mayo, 2018

Tipo de insulina	Sector público				Sector privado			
	Mediana de precio	RIQ	Precio mínimo	Precio máximo	Mediana de precio	RIQ	Precio mínimo	Precio máximo
**Humana**								
Regular	4,02	1,57	2,78	10,21	17,02	6,12	14,85	24,78
Isófana-NPH	4,08	1,34	3,40	12,56	14,70	6,16	3,09	22,57
Mixta humana	NA	NA	NA	NA	22,96	0,74	22,59	23,33
**Análoga**								
Rápida	NA	NA	NA	NA	46,20	11,35	40,72	56,72
Prolongada	60,16	32,87	32,87	64,20	70,03	30,36	49,01	107,73
Mixta análoga	NA	NA	NA	NA	53,07	NA	53,07	53,07

a Los precios se expresan en dólares estadounidenses (USD). Tipo de cambio al 8 de abril de 2018: 1 USD = 3232 PEN.

Otro mecanismo para disminuir el excesivo gasto en insulinas es procurar el uso de insulinas humanas y disminuir la preferencia de uso por insulinas análogas, al menos en el sector público (16). Es preciso señalar que el petitorio nacional de medicamentos esenciales no incluye en su listado insulinas análogas, hecho que se alinea a la recomendación del comité de expertos de la OMS que sugiere no incluir a las insulinas análogas en la lista de medicamentos esenciales (22). Por otra parte, tal como se muestra en nuestros resultados, los precios de las insulinas biosimilares resultan más económicos que los originales, pero aún existe controversia sobre su seguridad y eficacia (23). Perrin et al. sugieren que la armonización de la evaluación del *dossier* ayudaría a disminuir las barreras regulatorias y generaría confianza en el uso de insulinas biosimilares en los prescriptores y los pacientes (24).

Los hallazgos en este estudio pueden ser utilizados por los ministerios de salud para una visión general sobre la disponibilidad, precios y asequibilidad de los medicamentos que pertenecen a la lista de medicamentos esenciales y pueden servir como trazador del acceso a los medicamentos para la diabetes. Las diferencias de disponibilidad y precios entre sectores, regiones y niveles de atención es un hallazgo que demuestra que el acceso a medicamentos es inequitativo y es tarea del gobierno, junto al ministerio de salud, de monitorizar la disponibilidad y precios para luego idear planes para combatir estas inequidades. De igual manera, este trabajo puede ser útil para los clínicos, ya que se muestran algunos de los problemas con los que los pacientes se enfrentan cuando buscan sus medicamentos prescritos. Por último, estos resultados servirán a los pacientes para demostrar las limitaciones de acceso a las que se enfrentan en las seis regiones, realidad que probablemente es similar o más grave en otras regiones del Perú y, casi con certeza, similar a otros países de América Latina.

Este es uno de los pocos estudios reportados sobre precios de insulina realizados en América Latina con la metodología de OMS/AIS (16, 25), y es, tal vez, el primer estudio en el Perú que recolecta información sobre insulina y metformina. Sin embargo, es importante resaltar algunas limitaciones del estudio:

- Los resultados de disponibilidad corresponden al día de recolección de datos en cada establecimiento y no indica el promedio de disponibilidad en el tiempo. Sin embargo, dado que la encuesta se realizó en diferentes establecimientos en un período establecido, los resultados proveen una estimación cercana a la realidad que viven los pacientes en el día a día.
- Algunas regiones no fueron seleccionadas para la muestra porque contaban con cuatro hospitales o menos o el tiempo de acceso entre el hospital principal y los demás hospitales era mayor a tres horas. El no haber incluido a estas regiones puede haber sobreestimado los hallazgos de nuestro estudio, ya que es probable que estas regiones tengan menor disponibilidad.
- No se recogió información en las farmacias de hospitales privados, donde creemos la prescripción de insulinas análogas es mayor y, por ello, la disponibilidad puede ser más alta.
- Se obtuvo baja respuesta a solicitudes de los datos sobre las compras regionales o institucionales y esto podría ayudar a explicar la diferencia en los precios de compra de insulina por parte del paciente en los hospitales públicos.
- El número de establecimientos por región se apega a la metodología de OMS/AIS (12), que permite la comparabilidad con otros países, aunque no asegura la representatividad. Además, según la metodología, en algunas regiones fue necesario visitar farmacias de reserva para completar los datos de precio y disponibilidad, por lo que el número de establecimientos varía en cada región.
- Por último, en farmacias privadas, se encontraron farmacias cerradas al momento de la visita (20/184) y otras no aceptaron participar de la encuesta (43/184). Se recibió mayor rechazo de participación de farmacias de grupos comerciales (cadenas). Dado que no se recogieron datos de estos establecimientos, los análisis se realizaron con base en las 121 farmacias privadas en las que sí se recolectó información.

## CONCLUSIONES

Los problemas de disponibilidad, precio y asequibilidad de medicamentos para la diabetes, en particular de insulina, tienen múltiples causas. En farmacias públicas, es preciso mejorar los métodos de estimación de necesidades y supervisar cada proceso en la cadena de suministro de medicamentos, sobre todo en el abastecimiento descentralizado. Además, se deben monitorizar y procurar precios asequibles de los medicamentos para la diabetes, tanto en farmacias públicas como privadas.

### Agradecimientos.

Los autores agradecen a Farmacia Novofarma por permitirles hacer el piloto del estudio en sus instalaciones, al equipo de encuestadores que recolectó los datos, al Ministerio de Salud por autorizar la realización del estudio, y a los responsables de las direcciones regionales y al personal de las farmacias públicas y privadas por facilitar el acceso a la información.

### Financiamiento.

Stichting ICF como parte del estudio ACCISS “Adressing the Challenge and Constraints of Insulin Sources and Supply”.

### Contribución de los autores.

Todos los autores concibieron el estudio original y planificaron la recolección de datos. JTM y MLP analizaron los datos e interpretaron los resultados. JTM, MLP y LHP escribieron la primera versión del manuscrito. Todos los autores revisaron y aprobaron la versión final.

### Declaración.

Las opiniones expresadas en este manuscrito son únicamente responsabilidad de los autores y no reflejan necesariamente los de la *Revista Panamericana de Salud Pública* o la Organización Panamericana de la Salud.
